# Comparative transcriptome analysis reveals synergistic and disparate defense pathways in the leaves and roots of trifoliate orange (*Poncirus trifoliata*) autotetraploids with enhanced salt tolerance

**DOI:** 10.1038/s41438-020-0311-7

**Published:** 2020-06-01

**Authors:** Tonglu Wei, Yue Wang, Ji-Hong Liu

**Affiliations:** Key Laboratory of Horticultural Plant Biology (MOE), College of Horticulture and Forestry Sciences, Huazhong Agricultural University, Wuhan, 430070 China

**Keywords:** Abiotic, Plant physiology

## Abstract

Polyploid plants often exhibit enhanced stress tolerance relative to their diploid counterparts, but the physiological and molecular mechanisms of this enhanced stress tolerance remain largely unknown. In this study, we showed that autotetraploid trifoliate orange (*Poncirus trifoliata* (L.) Raf.) exhibited enhanced salt tolerance in comparison with diploid progenitors. Global transcriptome profiling of diploid and tetraploid plants with or without salt stress by RNA-seq revealed that the autotetraploids displayed specific enrichment of differentially expressed genes. Interestingly, the leaves and roots of tetraploids exhibited different expression patterns of a variety of upregulated genes. Genes related to plant hormone signal transduction were enriched in tetraploid leaves, whereas those associated with starch and sucrose metabolism and proline biosynthesis were enriched in roots. In addition, genes encoding different antioxidant enzymes were upregulated in the leaves (POD) and roots (APX) of tetraploids under salt stress. Consistently, the tetraploids accumulated higher levels of soluble sugars and proline but less ROS under salt stress compared to the diploids. Moreover, several genes encoding transcription factors were induced specifically or to higher levels in the tetraploids under salt stress. Collectively, this study demonstrates that the activation of various multifaceted defense systems in leaves and roots contributes to the enhanced salt tolerance of autotetraploids.

## Introduction

As sessile organisms, plants are constantly challenged by a range of adverse environmental factors, which are known as biotic (such as disease, insect and herbivores) or abiotic stresses (such as salt, cold, drought, heat, and flooding). Among them, high soil salinity is one of the major environmental constraints that has caused substantial yield and economic loss throughout the world^1^. Salt stress impedes plant growth and development, crop productivity and geographic distribution by imposing ionic toxicity and perturbing cellular osmotic potential due to excessive accumulation of Na^+ ^^2,3^. Therefore, it is pressing to understand the mechanisms underlying plant responses to salt stress and to create novel germplasms with enhanced salt tolerance that can be used to expand arable land and increase crop yield to meet the demands of feeding the growing world population.

There are several strategies to develop stress-tolerant crops that can maintain better growth and have stable yields under stressful conditions. Massive breeding efforts have been invested in cross hybridization, which has long been considered a major approach for obtaining germplasms with reinforced stress tolerance. Undoubtedly, this traditional breeding pipeline plays a crucial role in minimizing environmental influences on crop production and enabling sustainable agricultural production. However, it is worth mentioning that hybridization-based breeding is either time-consuming, unstable or even impossible for some plant species with reproductive barriers^4,5^. As an alternative, genetic engineering by manipulation of a variety of stress-responsive genes is now widely utilized as a modern breeding program to improve stress tolerance for a large spectrum of plants^6^. In addition, the selection of naturally stress-resistant plants (NSRPs) has recently been proposed to hold great potential for producing stress-tolerant germplasms that can be incorporated into conventional breeding platforms^5^. Apart from these strategies, ploidy breeding is increasingly emerging as an efficient method for providing desirable polyploid plants with improved tolerance to abiotic stresses^7–9^.

Polyploidy results from either genome duplication (autopolyploidy) or the combination of different genomes of related species^10^. Accumulating evidence suggests that polyploidization is a common phenomenon during the evolution of angiosperms and confers potential advantages in fitness under unfavorable environments^7,11–13^. Autopolyploids can be produced via chromosome doubling or through selection of the natural population in some specific plants, such as citrus^8,9,14^. Previous studies have demonstrated that polyploids display various responses to abiotic stresses relative to their diploid progenitors and that polyploids are generally more tolerant to abiotic stresses, including drought^9,15^, chilling^16^, and salt^17–19^.

During the past two decades, considerable progress has been made in elucidating plant responses to salt stress by using the excellent model plant *Arabidopsis thaliana* as the research organism. It is suggested that an array of salt-responsive functional or regulatory genes of plants may undergo extensive reprogramming, leading to physiological, biochemical and metabolic alterations to sustain the plant under harsh conditions^3^. To date, a variety of molecular machineries and the relevant signal networks involved in the salt stress response have been increasingly identified in a number of plant species, and the salt overly sensitive (SOS) signaling system, which plays a crucial role in regulating Na^+^ efflux under salt stress, is one of the best-characterized signaling cascades^3^. It has been well documented that modulation of ionic homeostasis, alleviation of osmotic stress, and mitigation of reactive oxygen species (ROS) accumulation are the major mechanisms responsible for combating ion toxicity and osmotic stress caused by salt stress^20^. Recently, there has been a significant breakthrough in understanding the salt-sensing mechanism of MOCA1, a glucuronosyltransferase for glycosyl inositol phosphorylceramide (GIPC) sphingolipids in the plasma membrane that has been described as a key component involved in adaption to various environmental salt levels^21^. However, it is worth mentioning that despite the abundance of sophisticated knowledge, the salt stress responses of polyploids relative to their diploid progenitors are largely unknown. In recent years, some studies have been carried out to investigate physiological or metabolic changes in polyploids under stresses^16,22–25^. Moreover, limited work has been performed to understand the molecular response of polyploids to stress, including analysis of transcriptional expression of stress-related genes^14,26,27^, genomic methylation and microRNA dynamics^17,28–30^. However, the underlying physiological and molecular mechanisms responsible for increased abiotic stress tolerance in polyploids remain largely elusive.

Citrus is one of the most important fruit crops worldwide, and citrus production is steadily increasing. Being predominantly subjected to vegetative propagation, citrus production is largely dependent on the rootstock used for grafting of scion cultivars. Trifoliate orange (*Poncirus trifoliata* (L.) Raf.) is widely used as a rootstock for the citrus industry due to its excellent traits, including cross compatibility with other related genera^31^ and superior resistance to cold stress and several diseases^32,33^. However, it is generally sensitive to salt^34,35^, limiting its application in regions with saline soils. Accordingly, a major goal for trifoliate orange improvement is to increase its salt tolerance. However, improvement through traditional hybridization breeding and genetic engineering has been difficult due to various reproductive factors, such as the long juvenile period, polyembryony, sterility, and recalcitrance to transformation and regeneration in vitro^5,6^. Given that polyploids may exhibit enhanced abiotic stress tolerance, increasing attention is being invested to explore trifoliate orange polyploids in an effort to generate novel germplasms with improved stress tolerance^9,14^.

In a previous study, we identified several naturally occurring autotetraploids among thousands of trifoliate orange seedlings^9^. Here, we further demonstrated that the tetraploids showed a drastic increase in salt tolerance relative to their diploid progenitors. We found that the leaves and roots of tetraploids showed distinct transcriptional responses to salt compared with those of diploids. Through analysis of the differences in gene expression and physiology between the tetraploids and diploids under salt stress, we conclude that enhanced salt tolerance in the tetraploids is attributed to a multifaceted action comprising altered hormone signaling and improved ROS scavenging ability in leaves and better ROS scavenging capacity and osmotic adjustment due to elevated accumulation of soluble sugar and proline in roots.

## Results

### Tetraploid plants show enhanced salt tolerance compared with diploid plants

**Fig. 1:**
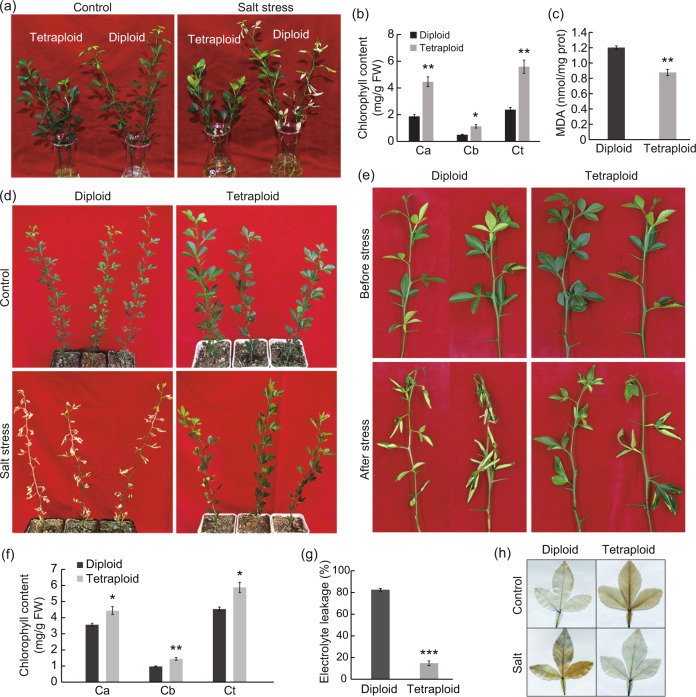
Trifoliate orange tetraploid plants display enhanced salt tolerance compared with diploid plants. **a**–**c** Phenotype (**a**), chlorophyll content (**b**) and MDA content (**c**) of three-month-old diploid and tetraploid plants after two weeks of culture in salt solution or water (control). **d** Phenotype of seven-month-old potted seedlings after 30 days of irrigation with salt solution or water (control). **e**–**g** Phenotype (**e**), chlorophyll content (**f**) and electrolyte leakage (**g**) of fresh shoots obtained from 2-year-old diploid and tetraploid plants before and/or after salt treatment. **h** Trypan Blue staining of diploid and tetraploid leaves with or without (control) salt treatment. Error bars indicate SE (*n* = 3). Asterisks indicate significant differences between the diploids and tetraploids (*P < 0.05; **P < 0.01; *** *P*< 0.001)

In an earlier study, we reported the identification of a number of autotetraploid plants from thousands of trifoliate orange seedlings. The autotetraploids showed some morphological changes, as shown by shorter plant stature and thicker leaves^9^. Here, efforts were made to assess the salt tolerance of tetraploid plants at three developmental ages. First, three-month-old tetraploid and diploid seedlings were subjected to hydroponic salt treatment (300 mM NaCl) for two weeks. There was no obvious difference in growth, development or physiology between the tetraploid and diploid plants when cultured in water only. In contrast, when plants were cultured in the salt solution, the leaves of the diploid plants showed more conspicuous wilting and yellowing compared with those of the tetraploids (Fig. 1a). Consistent with this phenotype, the chlorophyll content, including Ca, Cb, and Ct, in the diploid plants was lower than that in the tetraploid plants after salt treatment (Fig. 1b). The MDA content in the tetraploids was significantly lower than that in the diploids after salt stress, indicating that the tetraploids suffered less cell damage (Fig. 1c). Subsequently, seven-month-old tetraploid and diploid seedlings grown in soil were irrigated with 300 mM NaCl solution or water every four days. No obvious phenotypic difference between the diploid and tetraploid plants was observed when water irrigation was applied. However, when plants were irrigated with salt solution, the tetraploid plants displayed less serious damage than the diploid plants, and this damage was more apparent when the treatment duration was extended. After 25 d of salt stress imposition, severe necrotic symptoms were observed in the leaves of the diploids, whereas those of the tetraploids were healthier. When salt stress was prolonged to 30 d, the diploid plants became totally chlorotic, while most tetraploid plants remained green (Fig. 1d). Finally, fresh shoots detached from two-year-old diploid and tetraploid seedlings were placed in salt solution (300 mM NaCl) or water for 12 d. After the treatment, shoots from the diploid plants exhibited more serious wilting (Fig. 1e) and decreased chlorophyll content (Fig. 1f) compared with those from the tetraploid plants. In addition, the electrolyte leakage of tetraploids was significantly lower than that of diploids after salt treatment (Fig. 1g). Trypan Blue staining showed that leaves from the diploid plants displayed greater cell death relative to those from the tetraploid plants after salt stress (Fig. 1h). In addition, Na^+^ contents were measured from the leaves and roots of diploid and tetraploid plants without or with salt stress for 25 d. Surprisingly, we found that in the presence of salt stress, higher Na^+^ levels were detected in the two tissues, in particular the leaves, of tetraploids (Fig. S1). Taken together, these results revealed that the autotetraploid plants were more tolerant to salt stress than the diploid progenitors.

### Comparison of transcriptional profiling between tetraploids and diploids in response to salt stress

As a first step to exploring the underlying mechanisms of the enhanced salt tolerance observed in the tetraploid plants, we analyzed global transcriptional profiles of leaves and roots sampled from seven-month-old diploid and tetraploid plants treated with or without salt. For convenience of delineation, 2C and 4C were used to represent the diploid and tetraploid samples under control conditions, whereas 2S and 4S represent salt-treated diploid and tetraploid plants, respectively. We obtained ~200 Gb (gigabase) of quality-filtered sequence data, with >92.75% bases having a quality score of Q30 or higher (Table S1). Since the genome sequence of trifoliate orange is not available so far, the sequence reads were mapped to the pummelo (*Citrus grandis*) genome. We selected the pummelo genome for reference because it is the citrus genome with the best quality available^36^ and is closely related to trifoliate orange. In addition, a BUSCO analysis of the reference genome using 1375 eukaryotic genes in the Plantae BUSCO dataset demonstrated that 98.0% of the examined genes met the requirements, indicating that the assembly quality of the pummelo genome is high. Sequence mapping showed that the alignment rates ranged from 71.5 to 77.4% (Table S2).

**Fig. 2:**
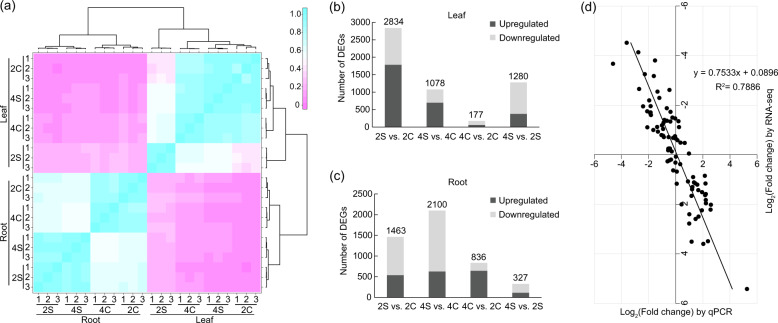
RNA-seq data and DEGs in diploids and tetraploids with or without salt treatment. **a** Hierarchical clustering of 24 samples based on the correlation coefficient (*r*^2^) between each sample. 2C and 4C are diploid and tetraploid, respectively, without salt treatment; 2S and 4S are diploid and tetraploid, respectively, under salt stress. The color panel represents the **r**^2^ values. **b**, **c** Statistics of up- and downregulated DEGs in leaves (**b**) and roots (**c**) for each pairwise comparison. Up- and downregulated DEGs are displayed in dark gray and light gray, respectively. **d** Correlation of expression changes observed by RNA-seq (*Y*-axis) and qPCR (*X*-axis)

We estimated expression values for all genes and performed hierarchical clustering for the samples based on the correlation coefficient, *r*^2^. As seen in Fig. 2a, root and leaf samples were clustered separately in the first clade, and three replicates for each treatment were clustered together in the final clade, indicating that gene expression profiles for the samples and replicates were highly consistent. To identify differentially expressed, salt-responsive genes in the roots and leaves between diploid and tetraploid plants, we subjected the expression values to pairwise comparisons, 2S vs. 2C, 4S vs. 4C, 4C vs. 2C and 4S vs. 2S (Tables S3 and S4). This analysis resulted in the identification of a total of 2834 genes that showed differential expression patterns in the salt-treated leaves of the diploid compared with the control (2S vs. 2C), whereas only 1078 differentially expressed genes (DEGs) were found in the tetraploid leaves under salt stress (4S vs. 4C, Fig. 2b). In contrast, in the root, there were 1463 DEGs in the salt-treated diploid (2S vs. 2C), which was less than those in the salt-treated tetraploid (4S vs. 4C; 2100). This finding implies that the responses of the leaves and roots of tetraploid plants to salt stress may vary at the transcriptional level. Additionally, 1280 DEGs were identified in the leaves when the diploid and tetraploid plants under salt stress were compared (4S vs. 2S), while only 327 DEGs were found in the roots (Fig. 2b, c). In the leaves, 379 genes were upregulated and 901 genes were downregulated in the tetraploid relative to the diploid under salt stress. For the roots, 112 and 213 genes were up- and downregulated, respectively, in the tetraploid in comparison with the diploid under salt stress (Fig. S2). To independently assess the reliability of the RNA-seq data, qPCR was used to analyze the expression patterns of 20 randomly selected genes. The correlation coefficient (*R*^2^ = 0.7886) between the qPCR and RNA-seq results was high, implying that the RNA-seq data are reliable (Fig. 2d). According to these analyses, we concluded that the global transcriptome in the tetraploids is altered when compared to the diploid ancestor.

### The stress-responsive genes of tetraploids are enriched under salt stress

**Fig. 3:**
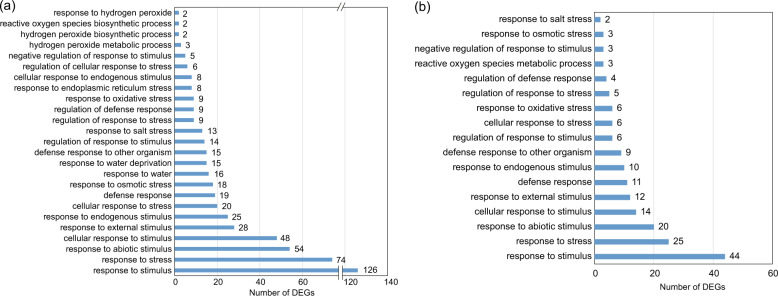
GO (gene ontology) analysis of DEGs. GO terms of the DEGs identified from tetraploids compared with diploids under salt stress (4S vs. 2S) in leaves (**a**) and roots (**b**). The numerals beside the histogram indicate the number of DEGs

To gain additional insight into the potential mechanisms that distinguish the responses of tetraploids and diploids to salt stress, we carried out a GO (gene ontology) enrichment analysis of the DEGs in both the leaves and roots of tetraploids relative to those of diploids under salt stress by comparing the transcriptomes of 4S and 2S (Tables S3 and S4). Several stress-related GO terms were commonly found in both leaves and roots, including ‘response to stimulus’, ‘response to stress’, ‘response to abiotic stimulus’, ‘cellular response to stimulus’, ‘defense response’, ‘response to osmotic stress’, and ‘response to salt stress’. Additional terms associated specifically with oxidative stress were also found, including ‘response to oxidative stress’, ‘hydrogen peroxide metabolic process’, ‘hydrogen peroxide biosynthetic process’, ‘reactive oxygen species biosynthetic process’, and ‘response to hydrogen peroxide’ in leaves and ‘response to oxidative stress’ and ‘reactive oxygen species metabolic process’ in roots (Fig. 3a, b). This result revealed that the transcriptional response to salt stress in tetraploids comprises increased numbers of stress-responsive genes relative to diploids.

### Hormonal signaling is altered in the leaves of tetraploids under salt stress

**Fig. 4:**
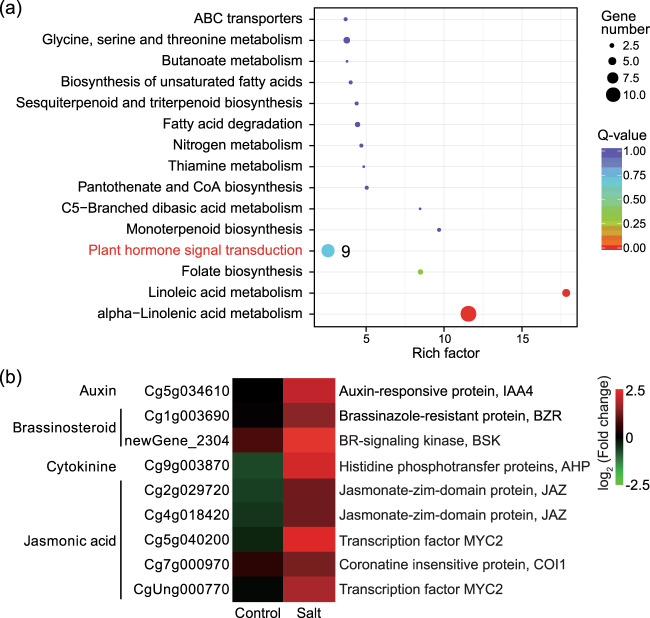
KEGG enrichment analysis of the DEGs in leaves and expression of plant hormone-related genes. **a** The 15 most significantly enriched KEGG pathways of upregulated genes in tetraploids relative to those in diploids under salt stress (4S vs. 2S). The dot color and size indicate the *Q*-value and gene number, respectively, as shown on the right. The numerals beside the dot indicate the number of DEGs enriched in the pathway. **b** Heatmap of gene expression for nine DEGs enriched in the ‘plant hormone signal transduction’ pathway. Heatmap color indicates fold change of expression in tetraploid leaves compared with that in diploid leaves under control and salt treatments

To identify metabolic pathways potentially associated with the enhanced salt tolerance of tetraploids, we carried out a standard pathway enrichment analysis based on Kyoto Encyclopedia of Genes and Genomes (KEGG). We analyzed the upregulated and downregulated genes in the tetraploid leaves relative to those in the diploid leaves under salt stress based on the pairwise assay (4S vs. 2S) and evaluated the 15 most significantly enriched pathways. The downregulated DEGs in the tetraploid were significantly enriched for ‘biosynthesis of amino acids’ and ‘phenylpropanoid biosynthesis’ (Fig. S3), while the upregulated DEGs were enriched for the ‘α-linolenic acid metabolism’ and ‘plant hormone signal transduction’ pathways (Fig. 4a). Considering that plant hormones have been shown to play vital roles in the abiotic stress response^37^, we focused on nine DEGs in the ‘plant hormone signal transduction’ pathway and compared their expression in tetraploids with that in diploids under control and salt conditions (Fig. 4b). These nine genes were related to the signaling network of auxin (IAA4), brassinosteroid (BZR, BSK), cytokinin (AHP) and jasmonic acid (two JAZ, two MYC2 and one COI1). We found that all these genes were upregulated in the tetraploid leaves compared with the diploid leaves under salt stress, implying that hormonal signaling was influenced in the tetraploid.

### Tetraploids accumulate more sugar and proline in the roots than diploids

**Fig. 5:**
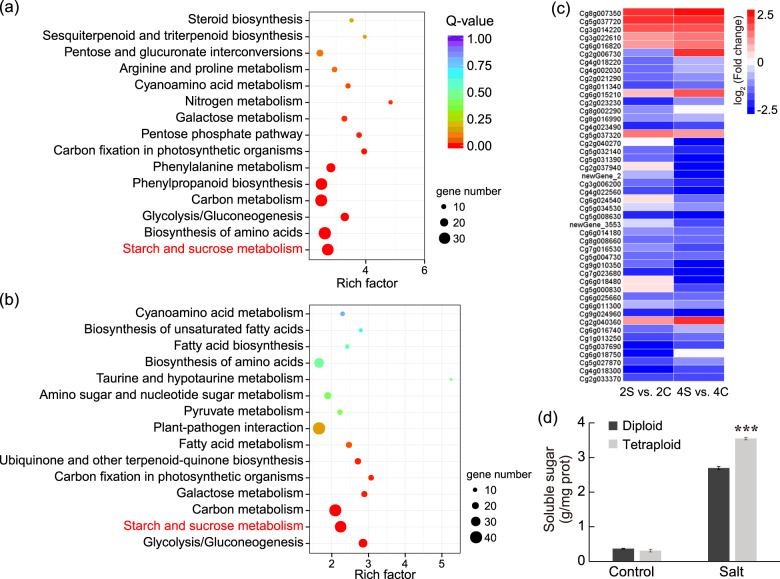
KEGG enrichment analysis of DEGs and soluble sugar content in diploid and tetraploid roots. **a**, **b** The 15 most significantly enriched KEGG pathways for DEGs in response to salt stress in diploids (**a**) and tetraploids (**b**). **c** Expression pattern of DEGs from the ‘starch and sucrose metabolism’ pathway in diploid and tetraploid roots under salt stress. Heatmap colors indicate fold change in expression, and red and blue indicate from high and low expression, respectively, as shown on the right. **d** Soluble sugar content in diploid and tetraploid roots under control and salt treatment. Error bars indicate SE (*n* = 3). Asterisks (***P < 0.001) indicate significant differences between diploids and tetraploids

To discern the differences in response to salt stress between the diploid and tetraploid roots, we also conducted KEGG enrichment analysis based on the DEGs found in 2S vs. 2C and 4S vs. 4C (Table S4). The 15 most significantly enriched pathways included ‘starch and sucrose metabolism’, ‘glycolysis/gluconeogenesis’ and ‘carbon metabolism’ for both diploid and tetraploid plants (Fig. 5a, b). Starch and sucrose metabolism determine the levels of soluble sugars and influence osmotic adjustment, which plays a vital role in salt tolerance^9,38^. Accordingly, we analyzed the gene expression patterns in the ‘starch and sucrose metabolism’ pathway in diploid and tetraploid roots in response to salt stress and found that the expression patterns of several genes implicated in these pathways in tetraploids were different from those in diploids in the presence of salt stress (Fig. 5c); this result indicates that salt-induced starch and sucrose metabolism in tetraploids might be different from that in diploids. To confirm this assumption, we measured the soluble sugar content in the diploid and tetraploid roots under control and salt stress and found that salt treatment led to a noticeable elevation in the sugar levels in both diploids and tetraploids. However, the tetraploids contained conspicuously higher levels of soluble sugars than the diploids (Fig. 5d).

**Fig. 6:**
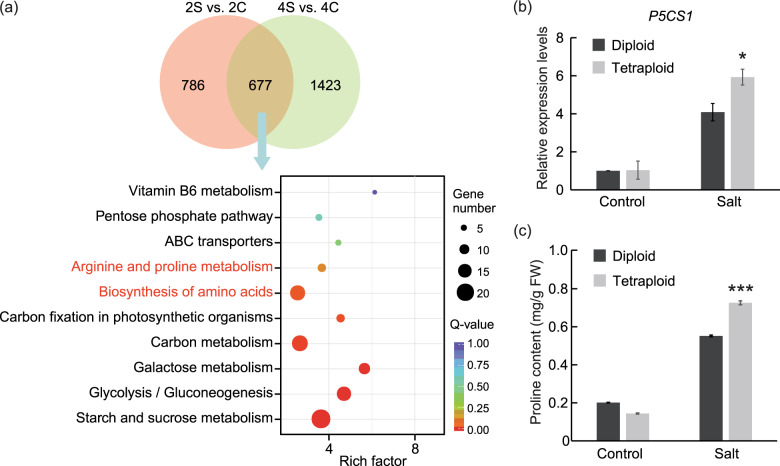
Expression patterns of proline-related DEGs and measurement of proline content in diploid and tetraploid roots under control and salt treatment. **a** Venn diagrams of DEGs in response to salt stress in diploids (2S vs. 2C) and tetraploids (4S vs. 4C) and the 10 most significantly enriched KEGG pathways based on the 677 DEGs. **b**, **c** Relative expression levels of the *P5CS1* gene (**b**) and proline content (**c**) in diploids and tetraploids under control and salt treatments. Error bars indicate SE (*n* = 3). Asterisks (*P < 0.05; ***P < 0.001) indicate significant differences between the diploids and tetraploids

Based on the DEGs in the roots revealed by comparing 2S vs. 2C and 4S vs. 4C (Table S4), we found that 677 genes were commonly influenced by salt in both diploid and tetraploid plants (Fig. 6a). KEGG enrichment analysis indicated that 10 pathways were most significantly enriched for DEGs involved in ‘Biosynthesis of amino acids’ and ‘Arginine and proline metabolism’, implying that accumulation of these amino acids might be relevant for the salt response of diploids and tetraploids (Fig. 6a). Previous studies have demonstrated that proline contributes to improved salt tolerance by acting as an osmoprotectant^2,39^. We found that a gene encoding ∆1-pyrroline-5-carboxylate synthase 1 (P5CS1), a rate-limiting enzyme for proline biosynthesis, was upregulated to higher levels in the tetraploid roots relative to the diploid roots in the presence of salt (Fig. 6b). Consistent with this, the tetraploids accumulated significantly greater amounts of proline under salt stress relative to the diploids (Fig. 6c).

### Tetraploids accumulate less ROS than diploids under salt stress

**Fig. 7:**
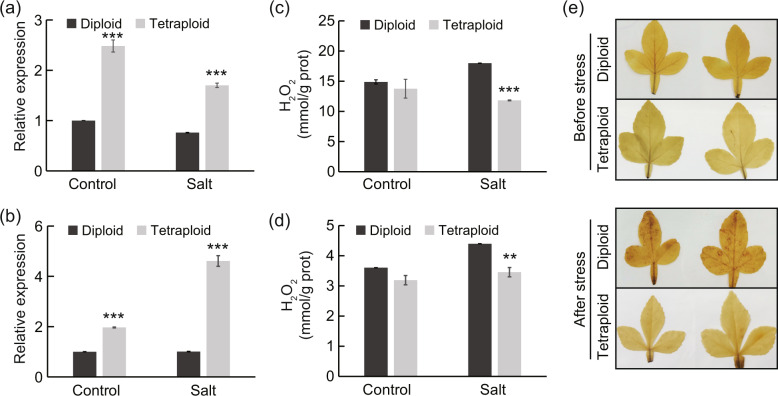
Antioxidant gene expression and ROS accumulation in diploids and tetraploids under control and salt treatment. **a**, **b** Relative gene expression levels of *POD* (Cg2g045100) in leaves (**a**) and *APX* (Cg3g014650) in roots (**b**) under control and salt treatment. **c**, **d** H_2_O_2_ content in the leaves (**c**) and roots (**d**) of diploid and tetraploid plants under control and salt treatment. Error bars indicate SE (*n* = 3). Asterisks indicate significant differences between the diploids and tetraploids (**P < 0.01; ***P < 0.001). **e** In situ visualization of H_2_O_2_ accumulation by DAB staining in diploid and tetraploid leaves before and after salt stress

Many abiotic stresses lead to increased levels of ROS, which may be detrimental to plant cells and organelles by causing oxidative stresses^40^. Interestingly, ROS and oxidative stress-related GO terms enriched among the DEGs were found to be differentially expressed between diploids and tetraploids under salt conditions. In particular, two antioxidant enzyme-encoding genes, *PEROXIDASE* (*POD*, Cg2g045100) and *L-ASCORBATE PEROXIDASE* (*APX*, Cg3g014650), displayed significantly higher expression levels in the leaves and roots of tetraploids, respectively, relative to diploids under salt conditions (Tables S3 and S4). Analysis with qPCR further confirmed the elevated transcript levels of these two genes in tetraploids (Fig. 7a, b). Moreover, we found that the leaves and roots of tetraploids accumulated lower levels of H_2_O_2_ in comparison with those of diploids, especially under salt conditions, as manifested by quantitative measurement and histochemical staining with DAB (Fig. 7c–e). Collectively, these results indicate that tetraploids have enhanced ROS scavenging capacity compared with diploids under salt stress.

### Transcription factors (TFs) are differentially expressed in tetraploids and diploids

**Fig. 8:**
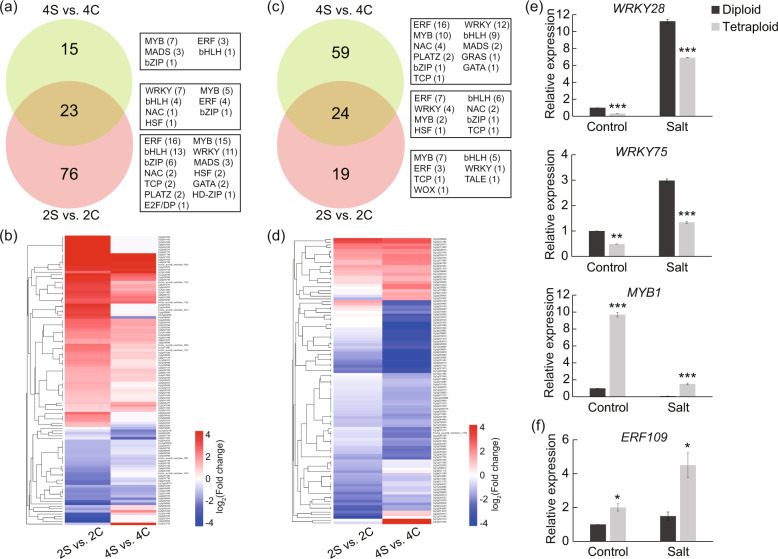
Analysis of differentially expressed TFs in diploids and tetraploids. **a**–**d** Venn diagrams and corresponding heatmaps for salt-induced TFs in the leaves (**a**, **b**) and roots (**c**, **d**) of diploids and tetraploids. The numerals inside the Venn circles indicate the number of differentially expressed TF genes in each comparison. The subfamilies are shown on the right, and the number of TFs in each subfamily is shown in parentheses. **e**, **f** Relative expression levels of three TF genes in leaves (**e**) and one TF gene in roots (**f**), as revealed by qPCR. Error bars indicate SE (*n* = 3). Asterisks indicate significant differences between the diploids and tetraploids (*P < 0.05; **P < 0.01; ***P < 0.001)

Transcription factors are known as master switches that function in multiple biological processes, including abiotic stress response, by regulating a range of downstream target genes. From our RNA-seq data, we found that many TF genes were prominently induced by salt treatment and showed different expression levels between diploids and tetraploids. For instance, a total of 23 TF genes, which mostly represented the WRKY, MYB, bHLH and ERF families, were induced by salt stress in the leaves of both diploids and tetraploids (Fig. 8a). In the tetraploids, 15 TF genes were specifically induced by salt, whereas 76 TF genes were specifically induced in the diploids (Fig. 8a, b). In the roots, 24 TFs were induced both in the diploids and tetraploids, whereas 59 and 19 TFs were specifically induced in the tetraploids and diploids, respectively, the majority of which are represented by the ERF, WRKY, MYB and bHLH families (Fig. 8c, d). We selected four genes, designated *WRKY28*, *WRKY75*, *MYB1*, and *ERF109*, for additional analysis. Both *WRKY28* and *WRKY75* were upregulated by salt and showed significantly lower expression in tetraploids than in diploids. In contrast, *MYB1* was downregulated by salt and showed significantly higher expression in tetraploids than in diploids (Fig. 8e). *ERF109* in roots was upregulated by salt, and the expression level of *ERF109* in tetraploids was obviously higher than that in diploids (Fig. 8f). These observations indicate that transcriptional regulatory mechanisms may be implicated in the enhanced salt tolerance of tetraploids.

## Discussion

In this study, we found that trifoliate orange tetraploid plants showed increased tolerance to salt stress compared with diploid progenitors. Our results are in agreement with earlier studies reporting enhanced tolerance to abiotic stresses in citrus polyploids^15,16,19,23,41^ or in other plants, including wheat^42^, rice^8^, Arabidopsis^43^, and black locust^25^. In a previous study, we reported that tetraploids displayed enhanced drought tolerance compared with diploids^9^. These findings seem to suggest that these tetraploids may exhibit simultaneous tolerance to multiple stresses, including salt and drought, which are two major environmental cues that drastically influence crop productivity and quality. Trifoliate orange is an important rootstock for citrus production, but vulnerability to salt and drought stress greatly impedes its application in regions where drought and salinity easily occur. The generation of trifoliate orange tetraploid plants with enhanced tolerance to both drought and salt is of agronomic significance. The tetraploids may hold great potential for rootstock breeding programs and germplasm enhancement if their agronomic traits meet the demands required for rootstocks. In the future, extensive work is necessary to evaluate these tetraploids to understand their potential for use as rootstocks.

Salt stress is known to cause ionic toxicity due to the accumulation of excessive detrimental ions, such as Na^+^. Therefore, plants may develop a sophisticated mechanism to exclude toxic ions to mitigate salt stress. Earlier studies have demonstrated that salt-tolerant genotypes may accumulate less Na^+^, which is a general rationale for combating salt stress^1–3^. Surprisingly, we found that in the present assay, significantly more Na^+^ was detected in the tetraploid plants than in the diploid plants. Although our result seems incongruent with many other studies indicating a positive correlation between enhanced salt tolerance and less Na^+^ accumulation, our finding is in line with an earlier work reporting that tetraploids presented a much greater accumulation of Na^+^ than did diploids^44^. The reasons behind this pattern of Na^+^ accumulation have not yet been determined; one reason may be the morphological differences in roots between diploids and tetraploids, leading to disparate hydraulic conductance and transpiration rates^23^. We thus assume that the tetraploid may hold a unique mechanism for buffering high Na^+^ levels, thus alleviating the negative impacts of Na^+^. However, the possibility that the tolerance of the tetraploid to salt stress is not dependent on overcoming Na^+^ accumulation cannot be fully ruled out. This hypothesis is not impossible, as chloride, but not Na^+^, has been considered to be a more toxic ion for citrus and trifoliate orange^23,44^, implying that the capacity for modulating chloride accumulation may play a role in salt tolerance. Therefore, efforts are required to assess chloride levels in diploids and tetraploids under salt stress in the future to understand the relationship between ion accumulation and salt tolerance in tetraploids.

In order to reveal the molecular mechanisms underlying improved stress tolerance, several previous studies have compared the transcriptomes between polyploids and diploids^14,26,27,45^. These studies generally concluded that whole-genome duplication has a strong and genome-wide effect on transcription and that the transcriptional response to stress varies between tetraploids and diploids^14,46,47^. In this study, we compared the transcriptomes of the roots and leaves of tetraploids and diploids under both nonstress and salt stress conditions. Our findings suggest that the transcriptome is altered globally in the two tissues of tetraploids. Strikingly, a limited number of DEGs were detected in the tetraploids under normal growth conditions, which may be explained by the fact that these tetraploids are of autopolyploid origin. However, the number of DEGs increased dramatically in the presence of salt stress, which underpins the importance of transcriptional reprogramming in the enhanced salt tolerance of polyploids. In addition, we noticed that the number of DEGs was smaller in the tetraploids in this study relative to the allotetraploids in an earlier study. However, the reasons for the transcriptional reprogramming of polyploids are still elusive and could be ascribed to multiple mechanisms. For example, methylation of transposable elements in autotetraploid rice was shown to affect gene expression^29^. In another work, microRNAs were suggested to play a critical role in the orchestration of gene expression in autopolyploid *Hordeum bulbosum*^17^. In the future, one of the challenges is to fully decipher the molecular mechanisms that account for the transcriptional change in tetraploids.

Plant hormones play important roles in growth and developmental processes as well as biotic and abiotic stress responses^20,35,48^. In this study, KEGG analysis of the upregulated DEGs revealed enriched pathways for ‘plant hormone signal transduction’, which included nine DEGs related to auxin, BR, cytokinin, and JA signaling pathways. Interestingly, the hormone signaling pathway was only identified in tetraploid leaves but not in tetraploid roots, which might be relevant to the phenotypic changes observed in the former, as most plant hormones have an impact on plant phenotypes^37^. Herein, we found that the genes involved in hormone signaling were upregulated to higher levels in tetraploid leaves compared with diploid leaves. It has been well documented that the signaling pathways of these four hormones play significant roles in the salt stress response^49–54^. This indicates that activation of hormone signaling might work as an indirect mechanism for enhanced salt tolerance by regulating other processes, such as growth and development. In addition, activation of these hormone signaling pathways suggests that crosstalk among them might possibly to make tetraploids better adapted to salt stress.

One of the adverse effects of excessive salt in plant tissues is osmotic stress; synthesis of compatible osmolytes, such as soluble sugars and proline, is critical for adjustment of osmotic potential under salt stress conditions^1,3^. Accumulating evidence indicates that enhanced production of soluble sugars, including sucrose, glucose, fructose, trehalose, and proline, in plant cells is conducive to increasing stress tolerance^2,39,55^. In this study, a *P5CS* gene involved in proline synthesis and several genes responsible for soluble sugar metabolism were induced to greater levels in tetraploids than in diploids in the presence of salt. Consistent with this transcriptional alteration, the roots of tetraploids contained significantly higher levels of proline and soluble sugars than those of diploids. Given the roles of proline and soluble sugars in plant salt tolerance, concomitant with our other findings, accumulation of more proline and soluble sugars may account, at least in part, for the enhanced salt tolerance of the tetraploids by facilitating the adjustment of osmotic potential in roots. This is reasonable, as roots are likely the first to be influenced by salt stress due to their direct submergence in the salt solution. As a consequence, the roots may thus accumulate osmoprotectants to cope with the stresses. However, it remains unclear by what mechanism the genes involved in proline and soluble sugar metabolism were upregulated to higher levels in tetraploids.

Oxidative stress is another effect of salt stress that promotes the generation of ROS^3^. It has been well documented that plants can activate antioxidant machineries comprising both enzymatic (POD, APX, SOD, and CAT) and nonenzymatic (ascorbic acid, glutathione, and nonprotein amino acids) pathways to alleviate ROS-induced damages^40^. It is conceivable that improving ROS scavenging capacity can significantly enhance the stress tolerance of plants^9,56,57^. In this study, we found that a *POD* gene in leaves and an *APX* gene in roots were upregulated, concomitant with less accumulation of ROS, in tetraploids compared with diploids, implying that the tetraploids exhibited more robust ROS scavenging ability in comparison with the diploids. This reveals that elevated antioxidant capacity contributes to the enhanced salt tolerance of tetraploids.

**Fig. 9:**
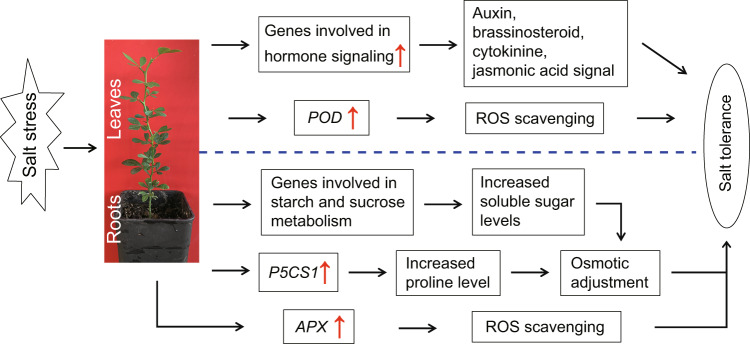
A model for mechanisms underlying the enhanced salt tolerance of trifoliate orange autotetraploids. Under salt stress, tetraploid plants exhibit enhanced salt tolerance in comparison with diploid progenitors due to the activation of multifaceted defense machinery in leaves and roots. In the leaves, activation of hormonal signaling and antioxidant enzyme (POD) genes leads to efficient hormonal signaling and improved *ROS* scavenging ability. In the roots, increased production of soluble sugars and proline due to upregulated expression of related genes, along with robust ROS scavenging ability by elevated expression of the *APX* gene, results in efficient adjustment of osmotic potential and detoxification of ROS

In summary, this study revealed that trifoliate orange tetraploids exhibited enhanced salt tolerance relative to diploid progenitors. Global transcriptome analysis allows us to propose a potential mechanism to explain the enhanced salt tolerance in tetraploids (Fig. 9). Upon exposure to salt stress, the tetraploid roots accumulate higher levels of sugar and proline, which function as compatible osmolytes, by upregulating genes involved in the metabolic pathway. As a result, osmotic stress to the roots can be mitigated to a greater extent in the tetraploid than in the diploid. On the other hand, tetraploid leaves undergo more extensive activation of hormone signaling pathways after sensing salt stress, which may trigger a range of defense machinery to counteract the salt stress. In addition, the tetraploids displayed a more powerful antioxidant system due to the higher expression level of antioxidant genes in leaves (*POD*) and roots (*APX*), allowing them to scavenge ROS in a more efficient manner in comparison with the diploids. In this regard, tetraploids may suffer from less serious oxidative stress and thus alleviate cell injuries under salt stress. Taken together, the current study revealed the physiological and molecular mechanisms underlying the enhanced salt tolerance of tetraploids and will help elucidate the mechanisms of polyploids in response to abiotic stresses.

## Materials and methods

### Plant materials and salt treatment

Autotetraploid and diploid trifoliate orange seedlings used in this study were described previously^9^. Plants were cultivated in the Citrus Germplasm Repository of Huazhong Agricultural University (Wuhan, China) under natural lighting and photoperiods. For salt stress treatments, three experiments were designed. In experiment I, three-month-old seedlings were grown hydroponically in 300 mM NaCl or water (control) for two weeks (the salt solution was changed every two days). In experiment II, seven-month-old potted plants were subjected to irrigation with 300 mM NaCl or water (control) every four days. In experiment III, fresh shoots were detached from two-year-old plants and placed in 300 mM NaCl or water (control) for 12 d. Each sample per treatment had at least three biological replicates, with one or two plants in each replicate, and stress treatment was repeated at least twice. Samples (leaves, roots) were collected before and after salt treatment and either used for physiological analysis (chlorophyll, electrolyte leakage, malondialdehyde, soluble sugar, proline, H_2_O_2_, and ROS staining) or immediately frozen in liquid nitrogen then stored at −80 °C for further analysis.

### Physiological measurements

Chlorophyll (a, b and total) content and electrolyte leakage (EL) were measured as previously described^9^. Malondialdehyde (MDA), soluble sugar, proline, and H_2_O_2_ were measured using commercial kits (Nanjing Jiancheng Bioengineering Institute, Nanjing, China) according to the manufacturer’s instructions. A spectrophotometer (UV-1800, Shimadzu, Japan) was used to measure absorbance values for these assays. Total protein content was measured with the Coomassie Brilliant Blue G-250 method^58^. Histochemical staining of H_2_O_2_ with DAB (3,3’-diaminobenzidine) was performed as previously described^38^. Cell death was evaluated by trypan blue staining according to a previous report^59^.

### RNA sequencing (RNA-seq) and data analysis

RNA-seq samples were collected from experiment II, in which diploid and tetraploid plants were sampled 25 days after salt treatment. Leaves and roots under control and salt treatment (three biological replicates, with two plants in each replicate; 24 samples in total) were sent to the Biomarker Technologies Company (Beijing, China) for RNA-seq. Total RNA was isolated using the RNAiso Plus Kit (TaKaRa, Dalian, China). RNA concentration and integrity were assessed on a NanoDrop 2000 (Thermo Scientific, MA, USA) using an RNA Nano 6000 Assay Kit (Agilent Technologies, CA, USA). Approximately 1 μg of RNA was used to construct cDNA libraries, and library quality was assessed on the Agilent Bioanalyzer 2100 system. The prepared libraries were sequenced on an Illumina HiSeq X Ten platform generating paired-end raw reads. After data processing, reads were filtered to remove adapters, reads containing poly(N), and low-quality reads. High-quality reads were mapped to a haploid pummelo (*Citrus grandis*) reference genome (http://citrus.hzau.edu.cn/orange/download/index.php) using HISAT2 with the parameter: “-p 6 -max-intronlen 5000000” (https://ccb.jhu.edu/software/hisat2/index.shtml). The assembly completeness of the reference genome was tested by BUSCO analysis^60^ using 1375 eukaryotic genes in the Plantae BUSCO dataset (http://busco.ezlab.org/v2/datasets), with the parameter “-m genome - sp arabidopsis”. Gene expression levels were calculated by the FPKM (fragments per kilobase of transcript per million fragments mapped) method. Differentially expressed genes (DEGs) were identified using the DESeq R package (Version 1.10.1; http://www.bioconductor.org/packages/release/bioc/html/DESeq.html) based on |log_2_ (fold change)| ≥1 and false discovery rate (FDR) < 0.01. GO (Gene Ontology) enrichment of the DEGs was performed by the GOseq R packages^61^. KEGG (Kyoto Encyclopedia of Genes and Genomes) pathway enrichment analysis was performed with KOBAS software^62^.

### Quantitative real-time reverse transcription PCR (qPCR) analysis

Total RNA used for qPCR was extracted as described above. First-strand cDNA was synthesized with a PrimeScript First-Strand cDNA Synthesis Kit (TaKaRa, Dalian, China) based on the manufacturer’s instructions. A total of 10 μL of reaction solution for qPCR analysis contained 100 ng of cDNA, 0.25 μM forward and reverse primer, and 5 μL of SYBR Green Master Mix (TaKaRa, Japan). The reaction program was composed of an initial denaturation step of 95 °C for 5 min, followed by 40 cycles of amplification at 95 °C for 10 s, 60 °C for 30 s and 72 °C for 15 s. The *ACTIN* gene of trifoliate orange was used as an internal reference. Gene-specific primers (Table S5) were designed using the Primer-Blast tool of NCBI (https://www.ncbi.nlm.nih.gov/tools/primer-blast/). Analysis of qPCR was performed on a QuantStudio 7 Flex system (CA, USA) according to the manufacturer’s protocol. The 2^-ΔΔCT^ method was used to calculate relative expression levels. Each sample had three technical replicates.

### Statistical analysis

Statistical data, shown as the means ± SE (standard error), were processed with Microsoft Office Excel. Statistical differences were determined using the one-way analysis of variance (ANOVA) method in SPSS (IBM, NY, USA) based on a *t*-test, with *P < 0.05, **P < 0.01 and ***P < 0.001 as significant.

## Supplementary information

Supplemental Figures

Supplemental Tables

## References

[1] Liang W, Ma X, Wan P, Liu L (2018). Plant salt-tolerance mechanism: a review. Biochem. Biophys Res. Commun..

[2] Dai W, Wang M, Gong X, Liu JH (2018). The transcription factor FcWRKY40 of *Fortunella crassifolia* functions positively in salt tolerance through modulation of ion homeostasis and proline biosynthesis by directly regulating *SOS2* and *P5CS1* homologs. New Phytol..

[3] Zhu JK (2016). Abiotic stress signaling and responses in plants. Cell.

[4] Dambier D (2011). Somatic hybridization for citrus rootstock breeding: an effective tool to solve some important issues of the Mediterranean citrus industry. Plant Cell Rep..

[5] Zhang S (2018). Reproduction in woody perennial Citrus: an update on nucellar embryony and self-incompatibility. Plant Reprod..

[6] Gong XQ, Liu JH (2013). Genetic transformation and genes for resistance to abiotic and biotic stresses in *Citrus* and its related genera. Plant Cell, Tissue Organ Cult..

[7] Comai L (2005). The advantages and disadvantages of being polyploid. Nat. Rev. Genet..

[8] Tu Y (2014). Genome duplication improves rice root resistance to salt stress. Rice.

[9] Wei T (2019). Enhanced ROS scavenging and sugar accumulation contribute to drought tolerance of naturally occurring autotetraploids in *Poncirus trifoliata*. Plant Biotechnol. J..

[10] Jiao Y (2011). Ancestral polyploidy in seed plants and angiosperms. Nature.

[11] Bird KA, VanBuren R, Puzey JR, Edger PP (2018). The causes and consequences of subgenome dominance in hybrids and recent polyploids. N. Phytologist.

[12] Parisod C, Holderegger R, Brochmann C (2010). Evolutionary consequences of autopolyploidy. New Phytol..

[13] Soltis PS, Soltis DE (2016). Ancient WGD events as drivers of key innovations in angiosperms. Curr. Opin. Plant Biol..

[14] Tan FQ (2015). Comparative metabolic and transcriptional analysis of a doubled diploid and its diploid citrus rootstock (*C. junos* cv. Ziyang xiangcheng) suggests its potential value for stress resistance improvement. BMC Plant Biol..

[15] Allario T (2013). Tetraploid Rangpur lime rootstock increases drought tolerance via enhanced constitutive root abscisic acid production. Plant Cell Environ..

[16] Oustric J (2017). Tetraploid Carrizo citrange rootstock (*Citrus sinensis* Osb.×*Poncirus trifoliata* L. Raf.) enhances natural chilling stress tolerance of common clementine (*Citrus clementina* Hort. ex Tan). J. Plant Physiol..

[17] Liu B, Sun G (2017). MicroRNAs contribute to enhanced salt adaptation of the autopolyploid *Hordeum bulbosum* compared with its diploid ancestor. Plant J..

[18] Meng HB (2011). Comparison between a tetraploid turnip and its diploid progenitor (*Brassica rapa* L.): the adaptation to salinity stress. Agric. Sci. China.

[19] Saleh B, Allario T, Dambier D, Ollitrault P, Morillon R (2008). Tetraploid citrus rootstocks are more tolerant to salt stress than diploid. Comptes Rendus Biologies.

[20] Yang Y, Guo Y (2018). Unraveling salt stress signaling in plants. J. Integr. Plant Biol..

[21] Jiang Z (2019). Plant cell-surface GIPC sphingolipids sense salt to trigger Ca^2+^ influx. Nature.

[22] Meng F (2016). Physiological and proteomic responses to salt stress in chloroplasts of diploid and tetraploid black locust (*Robinia pseudoacacia* L.). Sci. Rep..

[23] Ruiz M (2016). Tetraploidy enhances the ability to exclude chloride from leaves in carrizo citrange seedlings. J. Plant Physiol..

[24] Tan FQ (2017). Metabolic adaptation following genome doubling in citrus doubled diploids revealed by non-targeted metabolomics. Metabolomics.

[25] Wang Z, Wang M, Liu L, Meng F (2013). Physiological and proteomic responses of diploid and tetraploid black locust (*Robinia pseudoacacia* L.) subjected to salt stress. Int. J. Mol. Sci..

[26] Fasano C (2016). Transcriptome and metabolome of synthetic *Solanum* autotetraploids reveal key genomic stress events following polyploidization. N. Phytologist.

[27] Guo H (2017). Transcriptome analysis of neo-tetraploid rice reveals specific differential gene expressions associated with fertility and heterosis. Sci. Rep..

[28] Li X (2017). Analysis of small RNAs revealed differential expressions during pollen and embryo sac development in autotetraploid rice. BMC Genomics.

[29] Zhang J (2015). Autotetraploid rice methylome analysis reveals methylation variation of transposable elements and their effects on gene expression. Proc. Natl Acad. Sci. USA.

[30] Zhang HY (2016). Global methylation patterns and their relationship with gene expression and small RNA in rice lines with different ploidy. Front. Plant Sci..

[31] Rawat N (2017). Genome resequencing and transcriptome profiling reveal structural diversity and expression patterns of constitutive disease resistance genes in Huanglongbing-tolerant *Poncirus trifoliata* and its hybrids. Hortic. Res..

[32] Boava LP (2011). Global gene expression of *Poncirus trifoliata*, *Citrus sunki* and their hybrids under infection of *Phytophthora parasitica*. BMC Genomics.

[33] Şahin-Çevik M (2013). Identification and expression analysis of early cold-induced genes from cold-hardy *Citrus* relative *Poncirus trifoliata* (L.) Raf. Gene.

[34] Sykes SR (2011). Chloride and sodium excluding capacities of citrus rootstock germplasm introduced to Australia from the People’s Republic of China. Sci. Hortic..

[35] Kaleem F (2018). An overview of the genetics of plant response to salt stress: present status and the way forward. Appl. Biochem. Biotechnol..

[36] Wang X (2017). Genomic analyses of primitive, wild and cultivated citrus provide insights into asexual reproduction. Nat. Genet..

[37] Fahad S (2015). Phytohormones and plant responses to salinity stress: a review. Plant Growth Regul..

[38] Dahro B, Wang F, Peng T, Liu JH (2016). *PtrA/NINV*, an alkaline/neutral invertase gene of *Poncirus trifoliata*, confers enhanced tolerance to multiple abiotic stresses by modulating ROS levels and maintaining photosynthetic efficiency. BMC Plant Biol..

[39] Rady MM, Elrys AS, Abo El-Maati MF, Desoky ESM (2019). Interplaying roles of silicon and proline effectively improve salt and cadmium stress tolerance in *Phaseolus vulgaris* plant. Plant Physiol. Biochem..

[40] Choudhury FK, Rivero RM, Blumwald E, Mittler R (2017). Reactive oxygen species, abiotic stress and stress combination. Plant J..

[41] Balal RM (2017). Kinnow mandarin plants grafted on tetraploid rootstocks are more tolerant to Cr-toxicity than those grafted on its diploids one. Environ. Exp. Bot..

[42] Yang C (2014). Evolution of physiological responses to salt stress in hexaploid wheat. Proc. Natl Acad. Sci. USA.

[43] Chao DY (2013). Polyploids exhibit higher potassium uptake and salinity tolerance in *Arabidopsis*. Science.

[44] Mouhaya W (2010). Sensitivity to high salinity in tetraploid citrus seedlings increases with water availability and correlates with expression of candidate genes. Funct. Plant Biol..

[45] Zhang X, Deng M, Fan G (2014). Differential transcriptome analysis between *Paulownia fortunei* and its synthesized autopolyploid. Int. J. Mol. Sci..

[46] Dong Y (2017). Transcriptome-wide profiling and expression analysis of two accessions of *Paulownia australis* under salt stress. Tree Genet. Genomes.

[47] Xu E (2014). Transcriptome-wide profiling and expression analysis of diploid and autotetraploid *Paulownia tomentosa* × *Paulownia fortunei* under drought stress. PLoS One.

[48] Xia XJ (2015). Interplay between reactive oxygen species and hormones in the control of plant development and stress tolerance. J. Exp. Bot..

[49] Feng Y (2019). Natural variation in cytokinin maintenance improves salt tolerance in apple rootstocks. Plant, Cell Environ..

[50] Kagale S, Divi UK, Krochko JE, Keller WA, Krishna P (2007). Brassinosteroid confers tolerance in *Arabidopsis thaliana* and *Brassica napus* to a range of abiotic stresses. Planta.

[51] Khan M, Khan N (2013). Salicylic acid and jasmonates: approaches in abiotic stress tolerance. J. Plant Biochem. Biotechnol..

[52] Krishna P (2003). Brassinosteroid-mediated stress responses. J. Plant Growth Regul..

[53] Liu A (2019). Transcriptomic reprogramming in soybean seedlings under salt stress. Plant, Cell Environ..

[54] Yoon JY, Hamayun M, Lee S-K, Lee I-J (2009). Methyl jasmonate alleviated salinity stress in soybean. J. Crop Sci. Biotechnol..

[55] Mansour MMF, Ali EF (2017). Evaluation of proline functions in saline conditions. Phytochemistry.

[56] Gill SS, Tuteja N (2010). Reactive oxygen species and antioxidant machinery in abiotic stress tolerance in crop plants. Plant Physiol. Biochem..

[57] Zhang Q (2015). PtrABF of *Poncirus trifoliata* functions in dehydration tolerance by reducing stomatal density and maintaining reactive oxygen species homeostasis. J. Exp. Bot..

[58] Bradford MM (1976). A rapid and sensitive method for the quantitation of microgram quantities of protein utilizing the principle of protein-dye binding. Anal. Biochem..

[59] Pogány M (2009). Dual roles of reactive oxygen species and NADPH oxidase RBOHD in an Arabidopsis-*Alternaria* pathosystem. Plant Physiol..

[60] Simão FA, Waterhouse RM, Ioannidis P, Kriventseva EV, Zdobnov EM (2015). BUSCO: assessing genome assembly and annotation completeness with single-copy orthologs. Bioinformatics.

[61] Young MD, Wakefield MJ, Smyth GK, Oshlack A (2010). Gene ontology analysis for RNA-seq: accounting for selection bias. Genome Biol..

[62] Mao X, Cai T, Olyarchuk JG, Wei L (2005). Automated genome annotation and pathway identification using the KEGG Orthology (KO) as a controlled vocabulary. Bioinformatics.

