# Supplementary material for: Comparative transcriptome analysis reveals synergistic and disparate defense pathways in the leaves and roots of trifoliate orange (Poncirus trifoliata) autotetraploids with enhanced salt tolerance
Source: Hortic Res. 2020 Jun 1;7:88. doi: 10.1038/s41438-020-0311-7 (PMC7261775; doi:10.1038/s41438-020-0311-7)
Supplement: Supplementary file 1 — Supplemental Figures [file 41438_2020_311_MOESM1_ESM.doc]

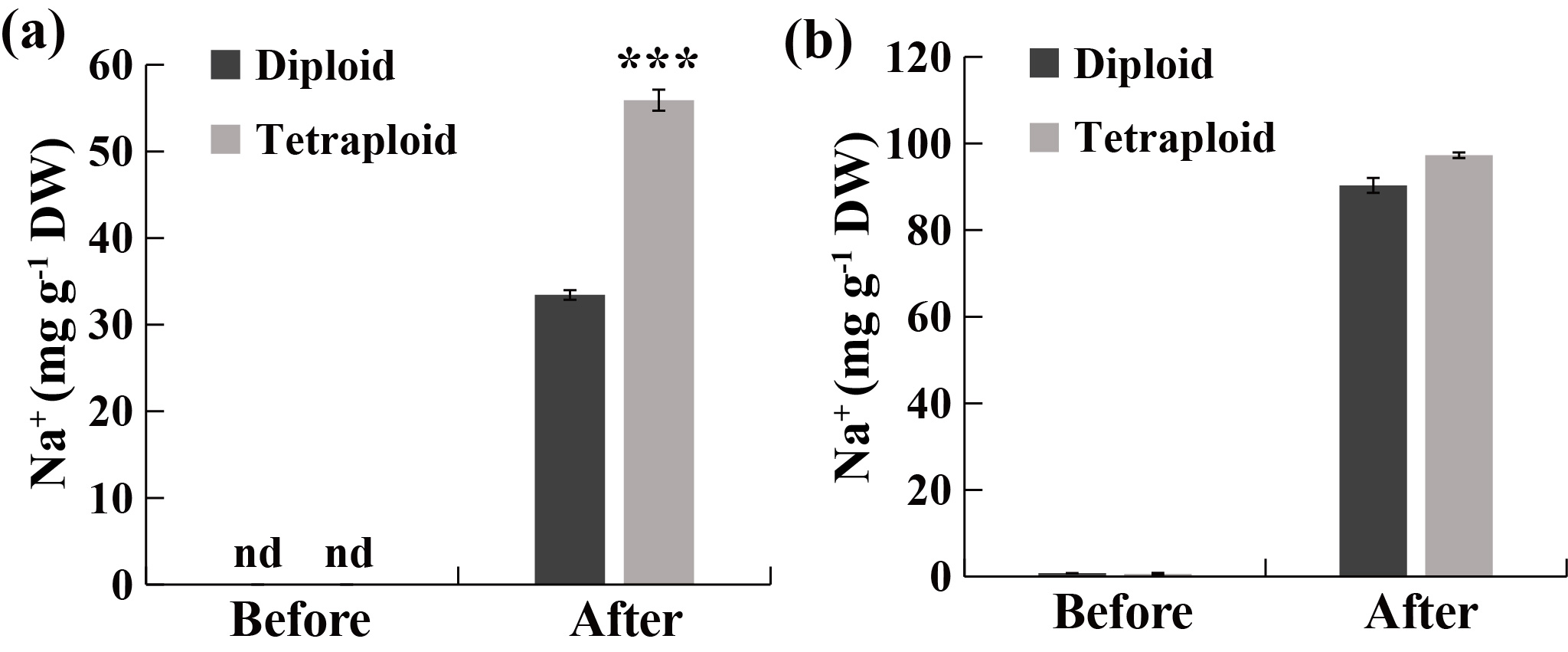


Fig. S1. Na^+^ content measured in the leaves (a) and roots (b) of the diploid and tetraploid plants before and 25 days after salt stress.


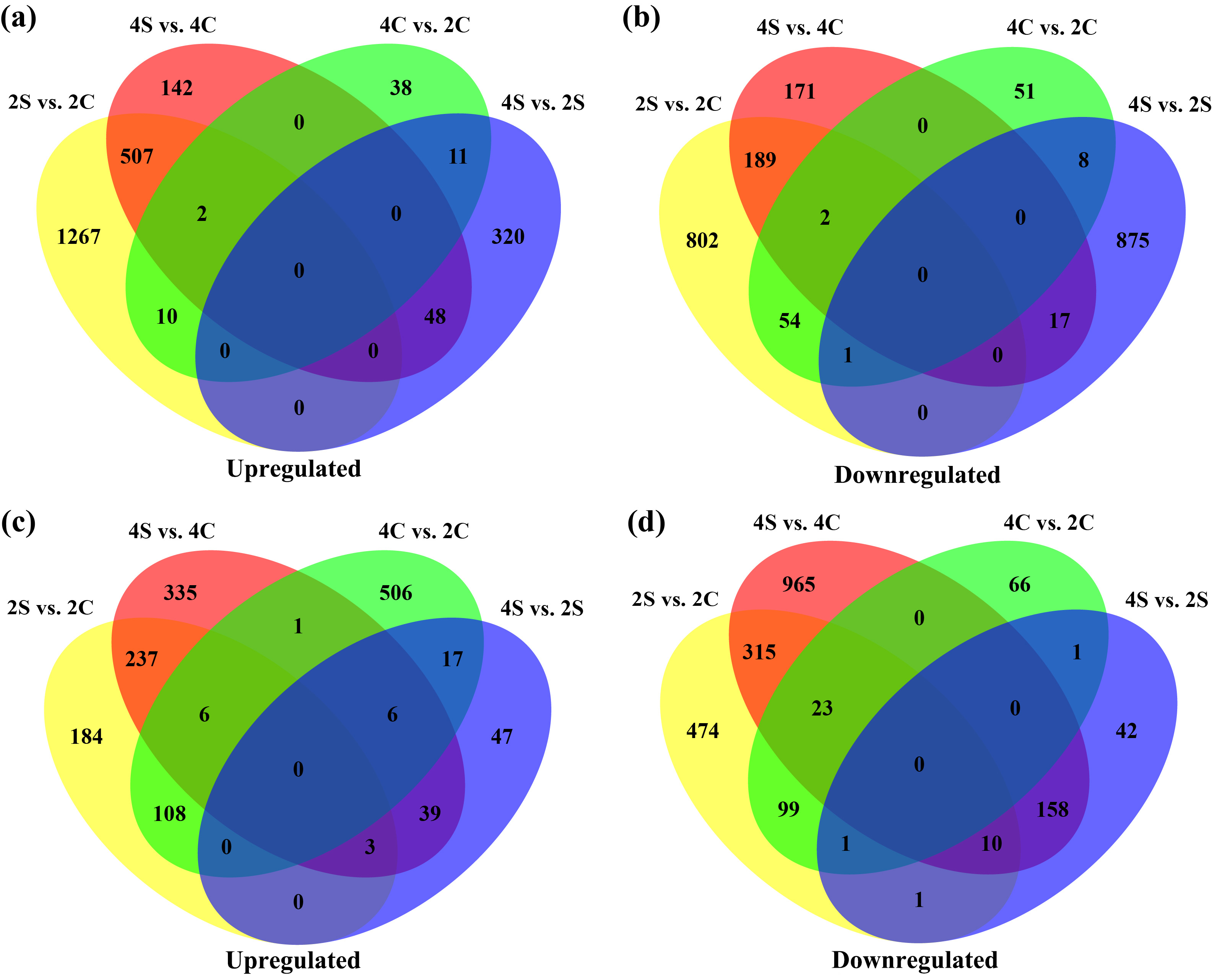


Fig. S2. Venn diagrams analysis of the upregulated and downregulated DEGs in different pairwise comparisons in two tissues, leaf (a, b) and root (c, d).


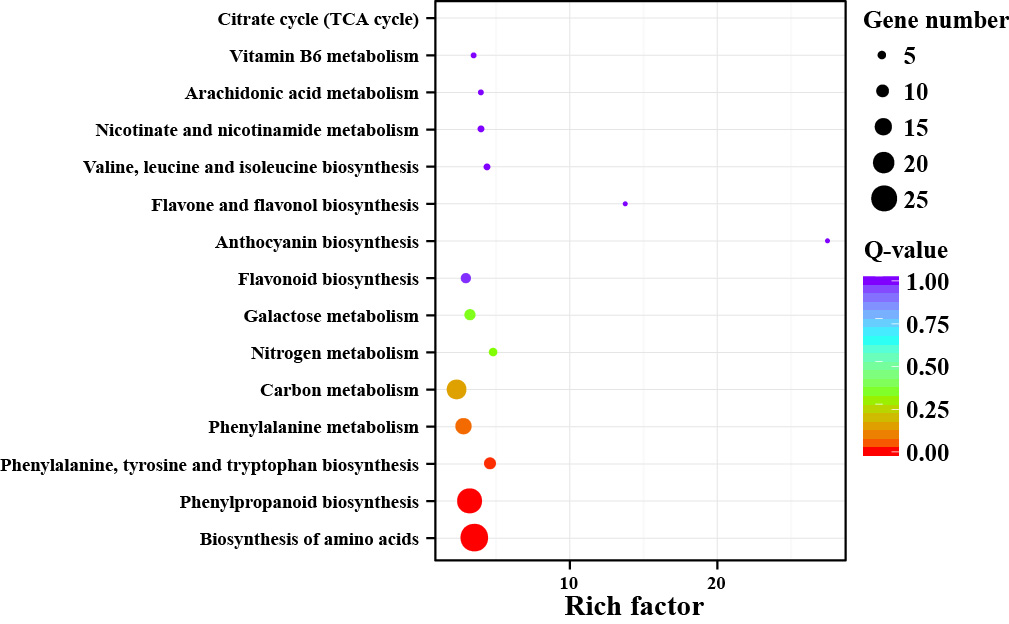


Fig. S3. KEGG analysis of downregulated DEGs in the leaves of tetraploids compared with those of diploids under salt stress (4S vs. 2S).
